# Supplementary material for: A sacrificial magnet concept for field dependent surface science studies
Source: MethodsX. 2022 Dec 9;10:101964. doi: 10.1016/j.mex.2022.101964 (PMC9791577; doi:10.1016/j.mex.2022.101964)
Supplement: Supplementary file 1 [file mmc1.docx]

**Supplementary material and Additional information:**

*Sample preparation*

We purchased commercial NdFeB magnets (supermagnete.ch: Q-10-03-02-HN) and wiped them with acetone and ethanol. The NbSe_2_ flake is taken from the same batch of crystals described in Ref. [4] that were grown by the chemical vapor transport method (ibid). We glue a single crystal of NbSe_2_ together with a cleaving post onto the sample holder, attach a magnet next to it, and transfer it without any further heat-treatment into our ultra-high vacuum system. Prior to transferring the sample into the 4 K cooled STM, we cleave the NbSe_2_ crystal in-vacuum at room temperature. For STM measurements, we use a tip made from electrochemically etched W that we treated by gently plunging it into an Au(111) sample until the apex is atomically sharp, which we verify by scanning across Au step edges. We record point and closed-loop spectroscopy using a conventional lock-in technique.

*Video Stills of the magnet insertion and sacrifice*

In Figure S1 we demonstrate the attachment of a NdFeB permanent magnet onto the sample plate next to the Cu_3_Au(111) crystal using the transfer tool, as shown in panel (a) and (b). The sacrifice of the magnet into the basked can be seen in panels (c) and (d), showing the heating of the sample and the magnet’s drop off into the collection basket.

*
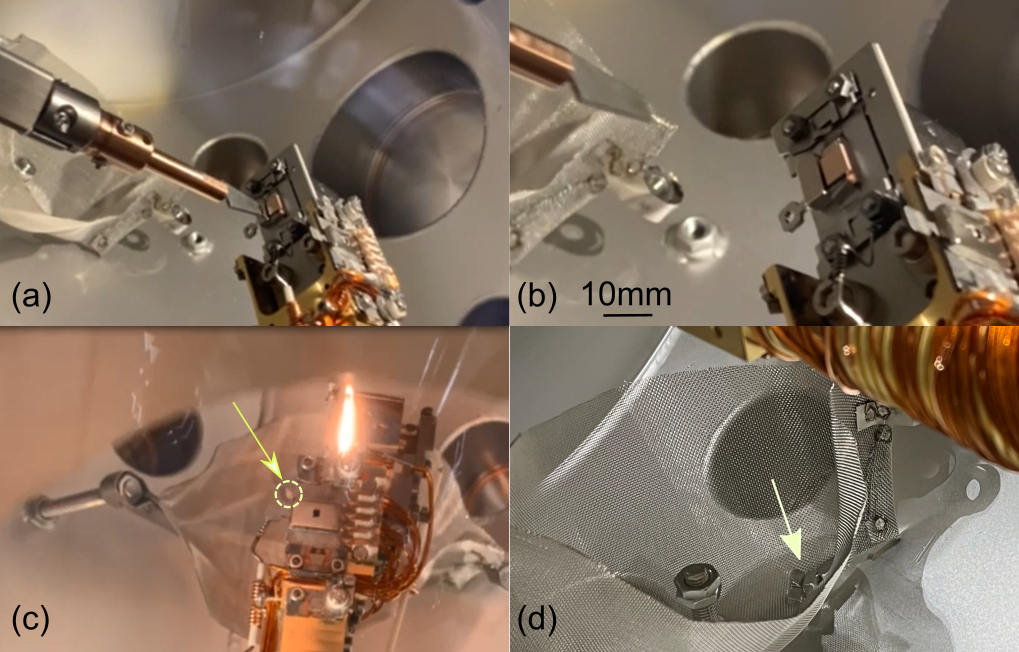
*

*Figure S1:* ***(a)*** *Permanent magnet attachment using the transfer tool.* ***(b)*** *Retraction of the transfer tool and attached magnet next to a Cu_3_Au crystal.* ***(c)*** *The magnet, as indicated by the dashed circle, drops by heating it above T*_C._ ***(d)*** *Collection basket with sacrificed magnets (arrow).*

*Determination of the distance L from the tip to the magnet edge*

*
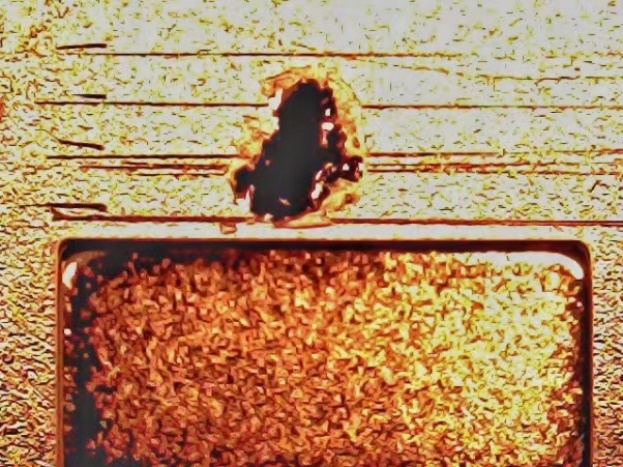
*

*Figure S2: Scratches on the sample holder help determine the tip position L with respect to the magnet edge. The long side of the magnet sets the scale to 10 mm.*
